# The Altered Somatic Brain Network in State Anxiety

**DOI:** 10.3389/fpsyt.2019.00465

**Published:** 2019-07-01

**Authors:** Xianrui Li, Meng Zhang, Kun Li, Feng Zou, Yufeng Wang, Xin Wu, Hongxing Zhang

**Affiliations:** ^1^School of Psychology, Xinxiang Medical University, Xinxiang, China; ^2^Department of Psychiatry, Henan Mental Hospital, The Second Affiliated Hospital of Xinxiang Medical University, Xinxiang, China; ^3^Henan key Laboratory of Biological Psychiatry, Xinxiang, China

**Keywords:** state anxiety, somatic marker hypothesis, somatosensory cortex, postcentral gyrus, resting-state functional magnetic resonance imaging

## Abstract

Highly anxious individuals often show excessive emotional arousal, somatic arousal, and characteristics of mental illness. Previous researches have extensively investigated the emotional and cognitive biases of individuals with high anxiety, but overlooked the spontaneous brain activity and functional connections associated with somatic arousal. In this study, we investigated the relationship between state anxiety and the spontaneous brain activity of the somatosensory cortex in a non-clinical healthy population with state anxiety. Furthermore, we also explored the functional connections of the somatosensory cortex. We found that state anxiety was positively correlated with the amplitude of low-frequency fluctuations (ALFFs) of somatic related brain regions, including the right postcentral gyrus (somatosensory cortex) and the right precentral gyrus (somatic motor cortex). Furthermore, we found that state anxiety was positively correlated with the connections between the postcentral gyrus and the left cerebellum gyrus, whereas state anxiety was negatively correlated with the connectivity between the postcentral gyrus and brain regions including the left inferior frontal cortex and left medial superior frontal cortex. These results revealed the association between the anxious individuals’ body-loop and state anxiety in a healthy population, which revealed the importance of somatic brain regions in anxiety symptoms and provided a new perspective on anxiety for further study.

## Introduction

Anxiety is a negative state of mind caused by stress or potential threat. It is a natural response to dangerous or threatening situations, which involves physical, affective, and cognitive changes (1, 2). During state anxiety attack, it is accompanied by obviously somatic symptoms. As a matter of fact, almost everyone will experience anxious emotion in their daily life, and existing research showed that the sub-threshold anxiety symptoms are highly detected in a non-clinical population (3). Previous studies have extensively explored the cognitive, emotional, and neural characteristics of anxious individuals (4, 5), overlooking the somatic brain network of anxious individuals.

The somatic marker hypothesis formulated by Antonio Damasio proposed that emotional processing guides behaviors, particularly decision-making (6). In Damasio’s model, all feelings are based on homeostatic representations of changes in the state of the body (7). This hypothesis also proposed that the somatosensory cortex (including postcentral gyrus) plays a crucial role in emotional processing and is usually activated by bodily states, and that substrates that represent visceral feeling states and interoceptive memories can influence decision-making (8).

Some evidence suggests that the postcentral gyrus is linked to anxiety disorders. Increased activity of the postcentral gyrus is primarily associated with decreased symptom of social anxiety (9, 10), whereas decreased activity of the postcentral gyrus is associated with symptoms of anorexia (11). Other studies revealed how the activity of the postcentral gyrus can be used to predict responses to treatment while processing happy facial expressions (12). The resting-state connectivity between the postcentral gyrus and the anterior cingulate cortex tends to correlate with poor emotional outcomes (13) and enhanced connectivity between the postcentral gyrus and the amygdala indicates a greater ability to regulate emotion (13). Although research findings support the role of the limbic system and the prefrontal cortex in anxiety disorders (14), the hypothesis proposed by Damasio, which focuses on bodily states, reminds us to pay more attention to somatosensory brain regions, especially the postcentral gyrus. Thus, it is important to identify the involvement of the postcentral gyrus in functional circuits underlying anxiety states.

Imaging researchers found a “fear network” that included the amygdala, anterior cingulate, and insula regions in patients with anxiety and anxiety-related disorder (15). Functional magnetic resonance imaging (fMRI) study results showed the hypofunction of prefrontal and anterior cingulate in patients with general anxiety disorder (16). A study in the patients with generalized anxiety disorder found the increased functional connectivity (FC) between the frontal and parietal, which is involved in the executive control network, and decreased FC between the insula and cingulate, which is in involved in salience network (17). Social anxiety was linked to the alterations of the bed nucleus of the stria terminalis (BNST), especially the across-effect of the BNST and the prefrontal (18). Additionally, an evidence has also showed the activity of the thalamus, parahippocampal gyrus, middle frontal gyrus, and inferior temporal gyrus in the high anxious group to be higher than that in the low anxious group (19). Although many researches have studied the changes of brain function in anxiety or anxiety-related disorders, the studies that enrolled a healthy population were still sparse.

We assumed that the variation in the brain function before suffering the disorder may be crucial for the early detection and intervention of the disease in the non-clinical population (20). The present study aimed to research how minor psychopathology is linked to brain functional in healthy adults. Based on the functions of the postcentral gyrus and the somatic marker hypothesis, we hypothesized that abnormal activity in the somatosensory cortex (including postcentral gyrus) would be related to the level of state anxiety and that this abnormal neural activity would be correlated with the mechanism underlying state anxiety. We used the amplitude of low-frequency fluctuations (ALFFs) to preliminarily study the association between state anxiety and the somatosensory cortex (especially postcentral gyrus). Furthermore, in order to determine the role of the postcentral gyrus in the functional circuits underlying state anxiety, we conducted correlational analysis between FC and state anxiety, which can reveal the association between the connection strength and state anxiety that share functional properties. This work’s aim is to research the relationship between the somatic brain regions and state anxiety, to highlight the significant role of the somatic regions and to give a contribution to further understanding anxiety.

## Materials and Methods

### Participants

A total of 138 participants were recruited from Xinxiang Medical University (28 males; mean age = 20.12, range = 17–25 years). All participants are right-handed. The participants were administered the Semi-Structured Clinical Interview of the *Diagnostic and Statistical Manual of Mental Disorders, Fourth Edition* (*DSM-IV*) by two experienced psychiatrists. The experimental methods were conducted in accordance with approved guidelines.

Anyone who met the axis I criteria of the DSM-IV was excluded, as well as individuals who could not stay motionless during the scan; had neurological diseases, serious psychiatric disorders, and head trauma; and were pregnant or breast-feeding. The state anxiety level of the 138 participants was assessed using the state anxiety inventory, and no one met the diagnostic criteria of anxiety disorder based on the *DSM-IV* according to the psychiatrists’ interview. Written informed consent was obtained from all the participants before the study, which was approved by the Institutional Human Participants Review Board of Xinxiang Medical University.

### Behavioral Assessments

State anxiety was measured with the State Anxiety Inventory (SAI), which is a self-report questionnaire that consists of 20 items. This scale has good internal consistency and test–retest reliability (21).

### Image Acquisition

#### T1 Data Acquisition

All the participants were scanned with a 3.0-T scanner (Trio Tim™, Siemens Medical Systems, and Erlangen, Germany) by a skilled technician. High-resolution T1-weighted 3D gradient-echo sequence images were obtained with the following parameters: field of view (FOV) = 256 ×256 mm^2^, resolution matrix = 256 × 192, slices = 128, thickness = 1.33 mm, repetition time (TR)/echo time (TE) = 2,530/3.39 ms, flip angle (FA) = 7°, voxel size = 1 × 1 × 1 mm^3^.

#### Resting-State Data Acquisition

All functional images were acquired with a 3.0-T scanner (Trio Tim™, Siemens Medical Systems, and Erlangen, Germany) by a skilled technician. Whole-brain resting-state functional images were acquired using a gradient-echo echoplanar imaging (EPI) sequence, with the following parameters: slices = 32; TR/TE = 2,000/30 ms; flip angle = 90°; field of view = 240 mm × 240 mm; thickness/slice gap = 3/1 mm; and matrix = 64 × 64, resulting in a voxel with 3.4 × 3.4 × 3 mm^3^. Two hundred forty functional images were acquired for each participant. Participants were instructed to close their eyes, stay awake, not to think of anything, and keep their head motionless during the entire scan; we also spoke to participants before and after the scanning sequence through the intercommunication device to prevent participants from falling asleep. A foam pillow was used to fixate the participant’s head to minimize involuntary head movements, and professional earplugs were used to reduce noise during the scan.

### Data Preprocessing

The resting-state image data were analyzed using Data Processing Assistant for Resting-State (DPARSF) software (22) (http://www.restfmri.net/forum/DPARSF). The first 10 images were discarded to eliminate the initial instability of the equipment, and the remaining 230 images were corrected for slice timing and head motions. Participants whose head motions exceeding 2 mm in translation or 2° in rotation during scanning were excluded from the study. Finally, 13 participants excluded from further study because translation and rotation exceeded ±2 mm or ±2°, and 125 participants were included in the succeeding study. We used Diffeomorphic Anatomical Registration Through Exponentiated Lie Algebra (DARTEL) to calculate the average template of the structures of all the subjects, calculated and stretched each structure after each participant’s segmentation, and then mapped the structures to the template. In order to generate the template, the program was continually refined until a better organizational template was generated and a gray template for the experiment was created. Subsequently, we standardized the registered gray-matter template and converted it to MNI (Montreal Neurological Institute) standard space. The functional images were normalized by using DARTEL. First, structural image was coregistered to the mean functional image after motion correction. Second, the transformed images were segmented into gray matter, white matter, and cerebrospinal fluid by using a unified segmentation algorithm; then, gray matter, white matter, and cerebrospinal fluid were used to create a template, and the motion-corrected functional volumes were spatially normalized to the MNI space using the normalization parameters estimated in DARTEL.

After spatial smoothing, whole brain signals, six head-movement parameters, white matter, and cerebrospinal fluid were removed or regressed to ensure that physiological noise had a minimal effect on the data. The Friston 24-parameter model (23) were used (6 motion parameters, 6 temporal derivatives, and their squares) to regress out head motion effects. We used a bandwidth of 0.01–0.08 Hz to filter the data and remove the effect of linear drift.

### Data Analysis

#### Amplitude of Low-Frequency Fluctuation

The ALFF measures the magnitude of regional activity amplitude, and it reflects the intensity of spontaneous regional brain activity. We used the DPARSF software to calculate the ALFF. The time series of the signal intensity of the whole brain was transformed into the frequency domain power spectrum by a Fourier transform, and the area under the peak of the power spectrum was regarded as the energy of the signal. Then, the square root was taken to represent the amplitude of the signal oscillation and the intensity of bold signal variation. In order to eliminate the difference in the overall level of whole-brain ALFF between individuals, the ALFF value of each individual was divided by the mean value of the whole-brain signals, and the whole-brain voxel was normalized to obtain the standardized ALFF graph.

#### Functional Connectivity

The right postcentral gyrus was taken as the seed region and the time series was extracted; then, Pearson’s correlation was used to analyze the association between the right postcentral gyrus time series and the whole-brain voxel time series. The *r* was converted to *z* using Fisher’s *z* transformation when calculating FC strength. FC was assessed by REST software (http://www.restfmri.net/forum/REST_V1.8).

## Statistical Analysis

Participants’ mean state anxiety scores were calculated using SPSS 20.0 (SPSS Inc., Chicago, IL, USA). We used SPM12 software to calculate the correlation between the ALFF and the state anxiety scores; the age, gender, and head motions were seen as covariates. A *P* < 0.05 with a cluster size of more than 45 was considered statistically significant. To determine the association between FC and the state anxiety scores, voxel-based multiple linear regressions were performed by SPM12, with age, gender, and head motions seen as covariates. A voxel level *P* < 0.005 with a cluster size of more than 30 was considered statistically significant.

## Results

### Clinical Variables

**Figure 1:**
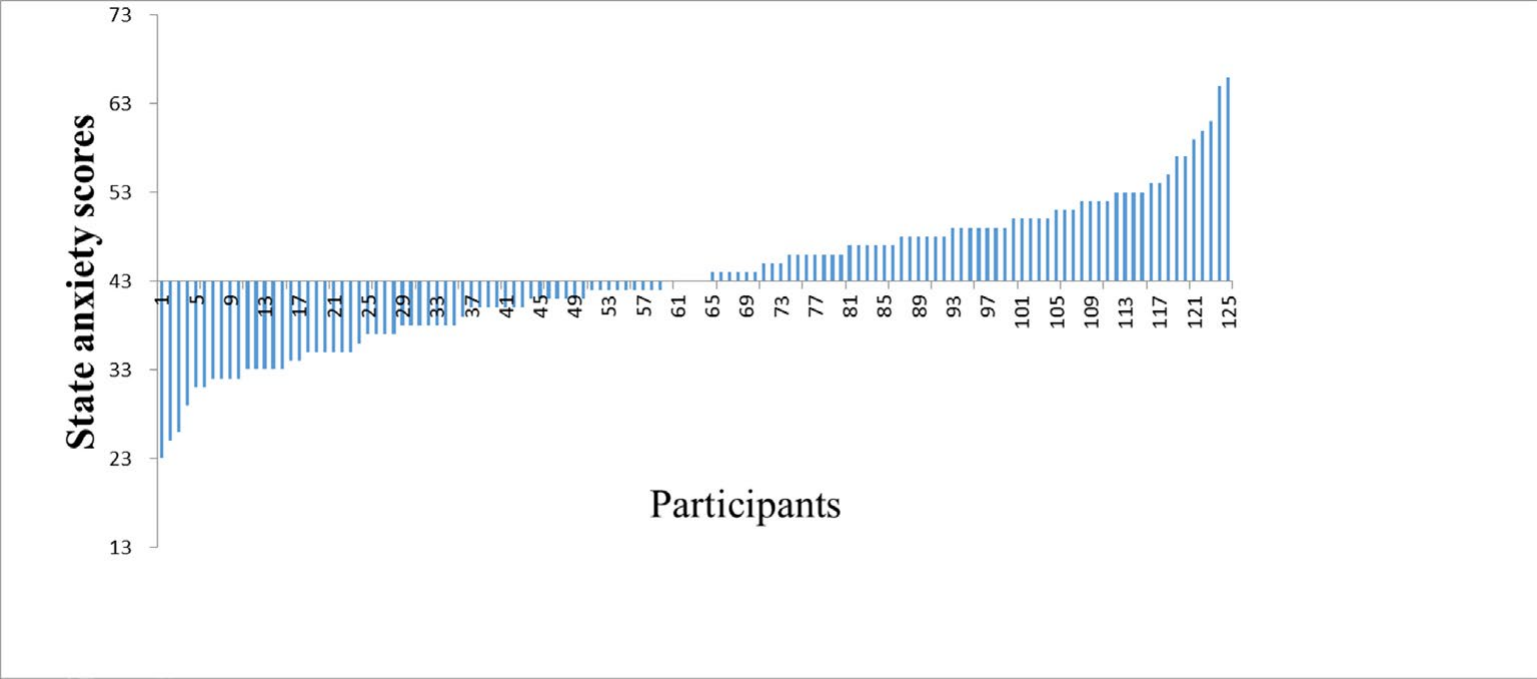
The distribution of the state anxiety scores in the healthy participants.

Finally, a total of 125 participants were enrolled in the study (22 males, mean age = 20.08, range = 17–25 years). The results of the study showed that the average score ± standard deviation of state anxiety was 43.57 ± 8.05 (see **Figure 1**).

### Correlations Between Amplitude of Low-Frequency Fluctuation and State Anxiety Scores

**Figure 2:**
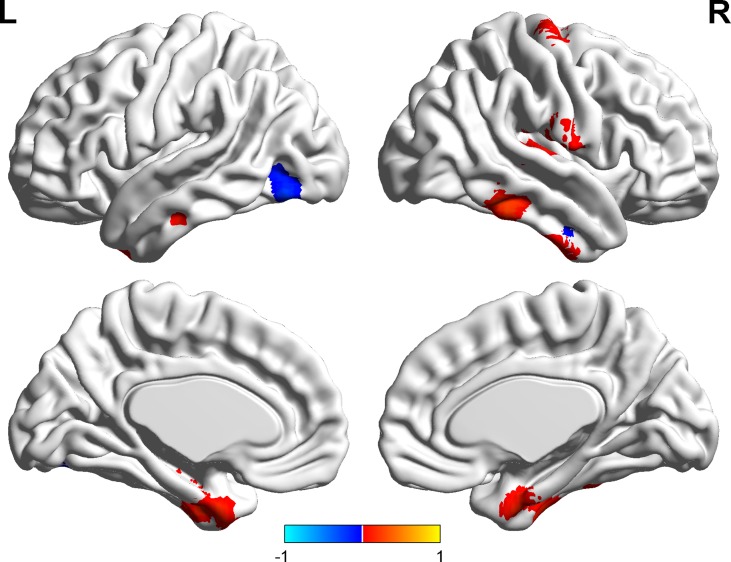
Brain areas with significant correlations between the amplitude of low-frequency fluctuation (ALFF) and state anxiety.

**Figure 3:**
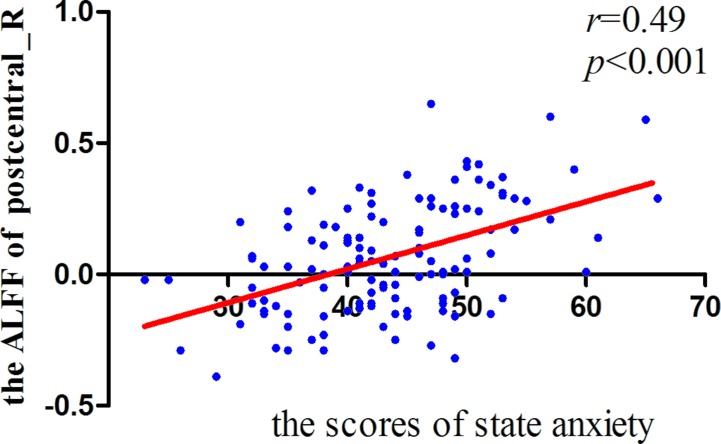
Correlation between right postcentral gyrus signals and state anxiety.

Significant correlations were found between the ALFF and state anxiety scores in the following brain areas. The ALFF values of the left inferior temporal cortex (MNI coordinates: −42, −9, −33; *r* = 0.43, *p* < 0.001), the right parahippocampal gyrus (MNI coordinates: 24, −3, −33; *r* = 0.40, *p* < 0.001), the right inferior temporal cortex (MNI coordinates: 60, −36, −21; *r* = 0.37, *p* < 0.001), the right postcentral gyrus (MNI coordinates: 54, −12, 18; *r* = 0.49, *p* < 0.001), and the right precentral gyrus (MNI coordinates: 21, −15, 72; *r* = 0.35, *p* < 0.001) were positively correlated with state anxiety. The ALFF value of the left inferior occipital cortex (MNI coordinate: −51, −69, −15; *r* = −0.38, *p* < 0.001) was negatively correlated with state anxiety (see **Table 1**; **Figures 2** and **3**).

**Table 1:** Brain regions with significant correlations between ALFF signals and state anxiety.

Brain regions	MNI coordinates			Voxel size	Peak values	r	p
	X	Y	Z				
Left inferior temporal cortex	−42	−9	−33	57	0.31	0.43	<0.001
Right parahippocampal gyrus	24	−3	−33	28	0.29	0.40	<0.001
Right inferior temporal cortex	60	−36	−21	68	0.36	0.37	<0.001
Right postcentral gyrus	54	−12	18	24	0.37	0.49	<0.001
Right precentral gyrus	21	−15	72	30	0.31	0.35	<0.001
Left inferior occipital cortex	−51	−69	−15	37	−0.35	−0.38	<0.001

### Correlations Between Functional Connectivity and State Anxiety Scores

**Figure 4:**
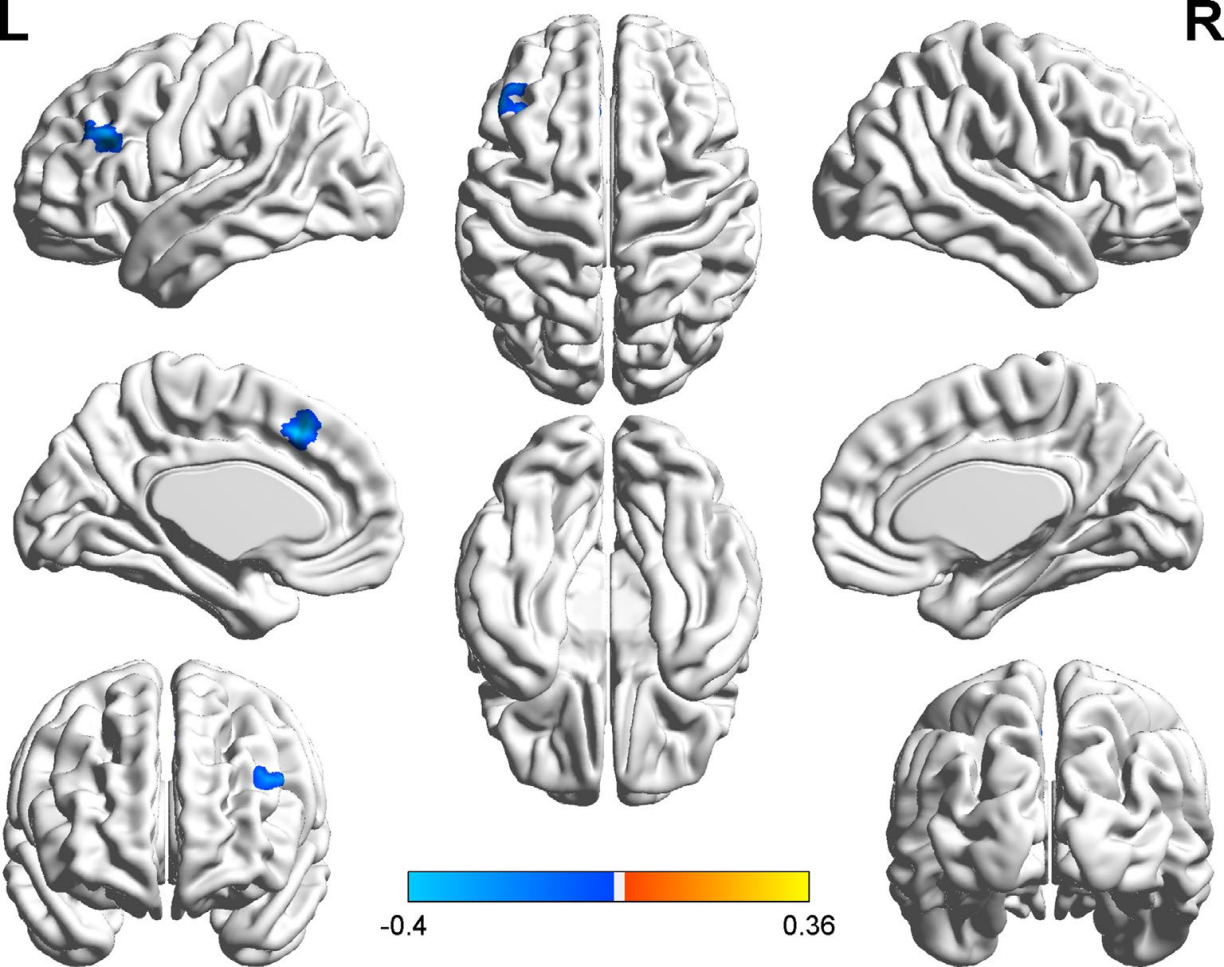
Brain regions with significant correlations between functional connectivity (FC) and state anxiety.

We explored the correlation between FC and state anxiety by using the right postcentral gyrus as a region of interest (ROI). The results showed that state anxiety was positively correlated with connectivity between the right postcentral gyrus and the left cerebellum gyrus (*r* = 0.43, *p* < 0.001) and that state anxiety was negatively correlated with connectivity between the right postcentral gyrus and the left inferior frontal cortex (*r* = –0.40, *p* < 0.001) and the left medial superior frontal cortex (*r* = –0.40, *p* < 0.001). Regarding the left inferior temporal cortex as ROI, state anxiety was negatively correlated with connectivity between the left inferior temporal cortex and the left middle temporal cortex (*r* = −0.46, *p* < 0.001), while regarding the right parahippocampal gyrus as ROI, state anxiety was positively correlated with connectivity between the right parahippocampal gyrus and the right precentral gyrus gyrus (*r* = 0.39, *p* < 0.001) (see **Table 2** and **Figure 4**).

**Table 2:** Brain regions with significant correlations between FC and state anxiety.

Brain regions	MNI coordinates			Voxel size	Peak values	r	p
	X	Y	Z				
Right postcentral as ROI							
Left cerebellum gyrus	−48	−57	−36	28	0.36	0.43	< 0.001
Left inferior frontal cortex	−48	27	27	27	−0.34	−0.40	< 0.001
Left medial superior frontal cortex	−6	21	45	20	−0.40	−0.40	< 0.001
Left inferior temporal cortex as ROI							
Left middle temporal cortex	−36	−54	12	29	−0.34	−0.46	< 0.001
Right parahippocampal gyrus as ROI							
Right precentral gyrus	51	−9	39	35	0.37	0.39	< 0.001

## Discussion

The aim of the current study was to highlight the key role of the somatosensory cortex (including postcentral gyrus) in healthy population with state anxiety by investigating the association between the spontaneous activity of postcentral gyrus and state anxiety as well as the relationship between FC regarding the right postcentral as ROI and state anxiety. The results showed that state anxiety was positively correlated with the spontaneous activity of the left inferior temporal cortex, the right parahippocampal gyrus, the right inferior temporal cortex, the right postcentral gyrus, and the right precentral gyrus measured with ALFF. These results suggest greater synchronization of these regions in relation to state anxiety. Furthermore, we found that state anxiety was negatively correlated with the left inferior occipital cortex.

Consistent with our hypothesis, the somatosensory cortex, especially the postcentral gyrus, which is a part of body-loop circuit, was found to play a critical role in anxiety, and that the spontaneous neural activity of the right postcentral gyrus was positively correlated with state anxiety. People with high state anxiety are usually more sensitive to daily events and stimuli; therefore, they are often in a high state of alertness and overreact to stimuli. One reasonable interpretation for the correlation between the postcentral gyrus and state anxiety is that a person in high state anxiety requires higher postcentral gyrus activity to cope with anxiety in order to prevent himself or herself from becoming pathologically anxious (24–26).

Some studies have demonstrated that one of the functions of the somatosensory regions is emotion perception (27, 28). On the anatomical level, the postcentral gyrus and its connections with frontal and parietal areas have been thought to be related to cognitive functions, such as control, memory, and attention (29). The postcentral gyrus is an important brain region for identifying basic emotions (30). It is involved in somatosensory processing and voluntary movement, and it is also active during emotion-regulation processing (31, 32). A study performed by Adolphs and Pourtois (33) demonstrated stimulating somatosensory cortex by transcranial magnetic stimulation can regulate emotion recognition in performing social face recognition task. A study of adolescents with anxiety showed that the difference in gray-matter volume of the postcentral gyrus is a crucial factor in a pathologic mechanism of anxiety disorders (34).

Besides the somatosensory cortex, we also found the activity in the bilateral inferior temporal cortex, the right parahippocampal gyrus, the right precentral gyrus, and the left inferior occipital cortex correlated with state anxiety. The functions of temporal lobe are linked to verbal expression and memory (35, 36). Processing happy facial expressions was related to the activation of the inferior temporal cortex (37), while when processing face emotion recognition tasks, temporal and parietal cortices showed higher activity in patients with general anxiety disorder than in healthy controls (38), which may indicate the insufficient ability in emotional working memory in the patient group. Existing study results showed the higher activation of the inferior temporal gyrus in the high anxious population compared to the low anxious population (19). Compared to healthy controls, the right middle temporal gyrus demonstrated higher activation in anxiety and mood patients with altered emotion regulation when performing cognitive reappraisal (39). Also, the hyper-gyrification in the right temporal gyrus was also found in general anxiety disorder patients, which reflects cortical folding during neurodevelopment (40). All in all, evidences suggest that the temporal lobe plays an important role in anxiety emotion, and the dysfunction of temporal cortices may be related to the poor cognitive function.

The parahippocampal gyrus is a part of the limbic system, which plays a vital role in memory processing. Emotional responses can be induced by the inner stimuli, such as autobiographical memory, and this memory is associated with the neuron responses of the parahippocampal gyrus (41). The emergence of unpleasant emotions usually increases the activity of the parahippocampal gyrus, and the repeated processing of aversive memories can cause chronic anxiety (42). Previous research found increased activity in the right parahippocampal gyrus of people with high anxiety compared to people with low anxiety (19). While processing the happy facial expressions, the parahippocampal gyrus showed increased activation compared to processing neutral facial expressions (37). Previous imaging research revealed the key role of the parahippocampal gyrus in anxiety, particularly linked to anxiety-related somatic complaints, and researchers found a correlation between the volume of parahippocampal and somatic complaints in non-clinical population (43). Moreover, the association between the volume of parahippocampal and negative automatic thoughts in healthy individuals was seen as an important factor to predict major depressive disorders (44), indicating the crucial role of the parahippocampal gyrus in mood or anxiety symptoms. It also showed that the parahippocampal gyrus and the inferior temporal cortex work together when processing and recognizing information (45), especially processing negative events. The precentral gyrus is mainly a motor region that is related to body movement, and some studies have showed that the activity of this region may influence emotional processing (46). As part of the core brain network, it is involved in distinguishing basic emotions (47). Studies have also found increased precentral gyrus activity in people with depression and anxiety disorders (39). The activity of the left inferior occipital cortex is negatively correlated with state anxiety, which may mean that people who have high state anxiety might use unconscious biases instead of physical properties to process visual stimuli (48).

It is important to explore how the postcentral gyrus contributes to state anxiety. Less negative connectivity in the postcentral gyrus may mean worse emotion regulation (13). In our study, we found that the connectivity of the right postcentral gyrus with the left cerebellum gyrus was positively correlated with state anxiety; the connections between the right postcentral gyrus and brain regions including the left inferior frontal cortex and the left medial superior frontal cortex were negatively correlated with state anxiety.

The cerebellum is necessary for basic cognitive processing and plays an important role in implicit learning and memory (49, 50). Previous research has demonstrated that the frontal lobe is associated with control functions (51), and evidence shows that, compared to low anxious individuals, anxious individuals exhibit weakened prefrontal control functioning in response to threat-related stimuli (51). The positive correlation between the strength of FC and state anxiety suggests that individuals with high state anxiety have heightened body-related monitoring and enhanced unconscious reactions. Our results showed that highly anxious individuals exhibited weaker correlations between the somatosensory cortex and the frontal lobe. This may indicate that during the initial stage of conscious processing, the frontal lobe may not be functionally coupled with the somatosensory cortex. Furthermore, while we regarded the left inferior temporal cortex as ROI, we found that the connectivity between the left inferior temporal cortex and the left middle temporal cortex was negatively correlated with the state anxiety. A study in macaque found that the inferior temporal cortex was modulated by facial expressions, which means that the inferior temporal cortex may be activated by the emotional expressions (52). The temporal lobe is responsible for some aspects of memory and emotion. Disorganized connectivity may be related to the memory and emotional dysfunction.

Regarding the right parahippocampal gyrus as ROI, the connectivity between the right parahippocampal gyrus and the right precentral gyrus was positively correlated with state anxiety. Considering that the activation in the precentral gyrus may be linked to emotional regulation (52), this result is not surprising; the parahippocampal gyrus may be seen as a functional hub that may represent increased integration between memory and emotion in processing negative events.

All in all, we found that the effect of state anxiety on the body eventually influenced the activity of the somatosensory cortex, especially the postcentral gyrus. The anxiety body-loop may explain the mechanism of anxiety to some extent, and abnormal activity in the postcentral gyrus may influence other behaviors, such as decision-making.

## Conclusion

People in the non-clinical population with high state anxiety have biases in emotional and cognitive processing that can make them more likely to suffer from somatic symptoms of anxiety compared to common people. However, the role of the body-loop in relation to state anxiety is not clear. Our research results demonstrated that the somatosensory cortex, especially the postcentral gyrus, plays a vital role in the mechanism of state anxiety using resting-state fMRI. Moreover, the present study should help us understand how the body-loop and the somatosensory cortex influence state anxiety.

## Limitation

We did not compare the differences in the body-loop of patients with anxiety disorder and healthy participants. Further studies are expected to combine different techniques to explore more information about state anxiety, and we intend to conduct further research based on the current results.

## Data Availability Statement

The datasets generated for this study are available on request to the corresponding author.

## Ethics Statement

This study was carried out in accordance with the recommendations of ethical requirements for biomedical research, Institutional Human Participants Review Board of Xinxiang Medical University, with written informed consent from all subjects. All subjects gave written informed consent in accordance with the Declaration of Helsinki. The protocol was approved by the Xinxiang Medical University Ethics Committee.

## Author Contributions

MZ, XW, and HZ developed the study concept and designed the study. XL, KL, FZ, and YW tested and collected the data. XL, KL, MZ, and XW analyzed and interpreted the data. XL and MZ drafted the manuscript and all authors revised it. All authors approved the final version of the manuscript for submission.

## Funding

This research was supported by the National Natural Science Foundation of China (31600927), the humanities and social science research Project of Henan Colleges and Universities (2017-ZZJH-422), the support project for the Disciplinary group of Psychology and Neuroscience, Xinxiang Medical University (2016PN-KFKT-28), the Science Fund for Distinguished Young Scholars of Henan (174100510024), the Program for Innovative Research Team (in Science and Technology) in University of Henan Province (18IRTSTHN025), and the Postgraduate research and innovation support program of Xinxiang Medical University (YJSCX201833Y).

## Conflict of Interest Statement

The authors declare that the research was conducted in the absence of any commercial or financial relationships that could be construed as a potential conflict of interest.
